# Group II Intron-Anchored Gene Deletion in *Clostridium*

**DOI:** 10.1371/journal.pone.0016693

**Published:** 2011-01-31

**Authors:** Kaizhi Jia, Yan Zhu, Yanping Zhang, Yin Li

**Affiliations:** Institute of Microbiology, Chinese Academy of Sciences, Beijing, China; National Institutes of Health, United States

## Abstract

*Clostridium* plays an important role in commercial and medical use, for which targeted gene deletion is difficult. We proposed an intron-anchored gene deletion approach for *Clostridium*, which combines the advantage of the group II intron “ClosTron” system and homologous recombination. In this approach, an intron carrying a fragment homologous to upstream or downstream of the target site was first inserted into the genome by retrotransposition, followed by homologous recombination, resulting in gene deletion. A functional unknown operon CAC1493–1494 located in the chromosome, and an operon *ctfAB* located in the megaplasmid of *C. acetobutylicum* DSM1731 were successfully deleted by using this approach, without leaving antibiotic marker in the genome. We therefore propose this approach can be used for targeted gene deletion in *Clostridium*. This approach might also be applicable for gene deletion in other bacterial species if group II intron retrotransposition system is established.

## Introduction

The genus *Clostridium* consists of over 100 species, ranking the second in size next to *Streptomyces* [1]. Many *Clostridium* species are closely related to human health. These include neurotoxigenic clostridia (*C. botulinum* and *C. tetani*) [2], clostridia involved in gas gangrene and necrotizing infections (*C. perfringens* and *C. sordellii*) [3], [4], [5], and the enteropathogenic *C. difficile* [6]. On the other hand, some *Clostridium* species are of great industrial importance. For example, *C. thermocellum* can produce ethanol from lignocellulosic waste at high temperature, while *C. acetobutylicum* and *C. beijerinckii* produce solvents (acetone, butanol, and ethanol) by utilizing a variety of substrates from monosaccharides to polysaccharides [7], [8], [9]. In view of the importance of *Clostridium*, it is desirable to understand both the virulence mechanism of pathogenic *Clostridium* and the industrial characteristics of *Clostridium* used in fermentation industry.

The virulence mechanism and the desirable industrial characteristics of clostridial strains are usually controlled by many genes [3], [4], [5], [10], [11]. Therefore, a systematic approach to understanding or engineering these strains often requires manipulating multiple genes [10]. Targeted inactivation of clostridial genes can be achieved by Campbell-like integration through homologous recombination of a replication-defective plasmid. Successful applications have been reported in *C. acetobutylicum* [12], [13], [14], [15], *C. beijerinckii* [16], *C. perfringens* [15], [17], [18], [19], and *C. difficile* [16], [20], but their transformation frequency was around 10^−3^, and usually one single-crossover integrant can be obtained from 1 mg plasmid DNA, suggesting the integration efficiency was very low. In addition, these single-crossover events are segregationally unstable [12], [13], [14], [15], [16], [17], [18], [19], [20]. Antisense RNA technology has also been applied in *Clostridium*, such as the downregulation of butyrate kinase and coenzyme A transferase in *C. acetobutylicum* [21], [22]. However, as the antisense RNA might affect the cell transcriptional program, the phenotypic changes might not be directly related to the downregulation of target genes [21], [23]. Recently, a gene knockout system that employees the *Lactococcus lactis* Ll.LtrB group II intron has been developed and adapted for directed insertional gene inactivation in *Clostridium*, which is termed as “ClosTron” [24]. To date, it has been applied in *C. acetobutylicum*, *C. difficile*, *C. beijerinckii*, *C. botulinum*, *C. sporogenes* and *C. perfringens* [1], [6], [24], [25], [26], [27], [28], [29], [30]. In particular, it should be noted that *C. difficile* was almost refractory to mutagenesis until the development of the ClosTron mutagenesis system [31]. This system involves homing of ribonucleoprotein (RNP) complex that consists of group II intron RNA molecule (Ll.LtrB) and the associated protein LtrA [32]. Base pairing with the DNA insertion site in the first place and then catalyzing insertion and reverse transcription of the intron RNA by RNP confer specificity upon the subsequent integration event [32]. To achieve rapid and efficient selection of positive integrants, a retrotransposition-activated selectable marker (RAM) was introduced into intron domain IV (DIV) [33]. However, this strategy cannot be used to isolate clones containing a second intron insertion in an already erythromycin-resistant mutant. To solve this problem, RAM was flanked by two repeated FLP (flippase) recognition target (FRT) sites and it can be removed from the chromosome in FLP recombinase-mediated step [30]. Therefore, the authors proposed that this system could be applied for insertional mutation of multiple gene in *Clostridium* [30]. Nevertheless, this approach would leave an intron residual fragment of over 0.9 kb in the genome [30]. The disruption of the other genes by this system in an already intron insertion mutant might lead to the instability of the previously mutated genes due to the presence of LtrA, through which the excision of a DNA sequence flanked by two intron fragments might occur via homologous recombination [1], [24], [34], [35]. So far, there has no report using the above described strategy for gene deletion in *Clostridium* yet.

To date, *C. acetobutylicum* and *C. thermocellum* are the only clostridial strains in which targeted gene deletion via homologous recombination have been established [36], [37]. In *C. acetobutylicum*, *spo0A* was deleted by using a replicative plasmid pETSPO capable of integrating into the chromosome through two rounds of crossover selection [36]. pETSPO contains a Gram-positive origin of replication and was methylated before introducing into *C. acetobutylicum*, which significantly increased the chance of homologous recombination [36]. Nevertheless, follow-up researchers suggested that this method is of low reproducibility and laborious to screen for double-crossover integration events [34], [38]. Moreover, this strategy would leave an erythromycin resistance marker in the genome for screening, which prevents it from manipulating multiple genes, since not many markers are available for *Clostridia* [21], [36], [39], [40], [41], [42], [43], [44]. In *C. thermocellum*, a replicating allelic exchange vector was also adopted to delete targeted genes because the current transformation efficiency does not meet the requirement for genetic manipulation; therefore this method should be laborious to screen for double-crossover integration events [37].

The aim of this study was to develop a more efficient targeted gene deletion strategy for *Clostridium*. By combining the principles of the “ClosTron” system and homologous recombination, we developed an accurate gene deletion procedure which enabled us efficiently delete DNA fragments without leaving any antibiotic resistance marker in the genome. This strategy might aid in the genetic dissection of clostridial virulence and engineering industrial clostridial strains for efficient production of biofuels and bio-based chemicals.

## Results

### The strategy to delete target gene in *Clostridia*

**Figure 1:**
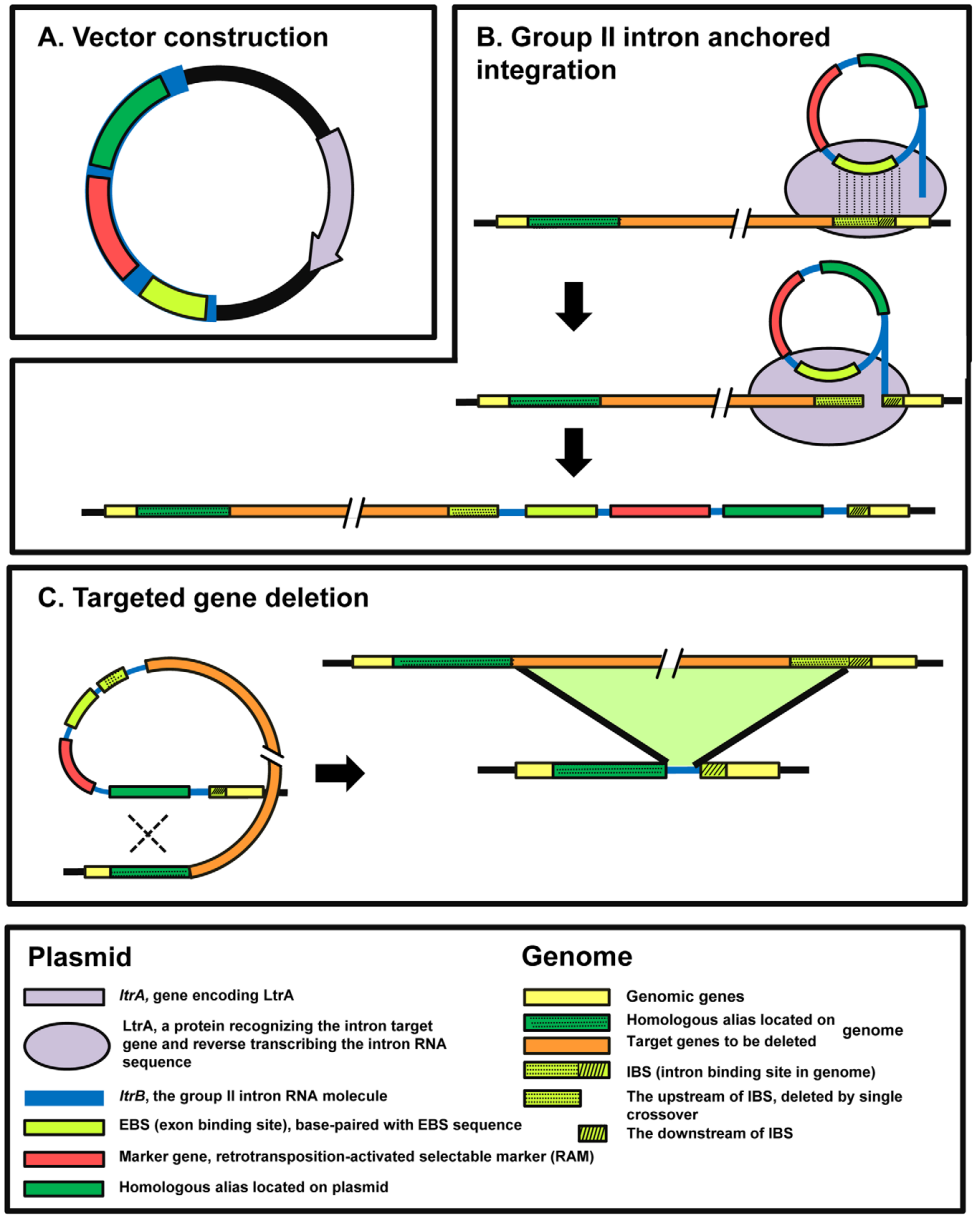
The strategy for target gene deletion in clostridial genome.

In microorganisms, gene deletion was usually conducted by using the classical homologous recombination strategy. Although single crossover integration can inactivate the target genes in *Clostridium*, the transformation and recombination frequencies were so low that double crossover events rarely occurred [45]. Targeted gene deletion via double crossover recombination remains a challenge for *Clostridium* [24], [36], [46]. ClosTron has been adopted for targeted gene disruption in *Clostridium*, with an integration frequency of nearly 100% in some clostridial species [24]. We therefore propose an intron-anchored gene deletion approach (Fig. 1). In this approach, an allele homologous to the upstream or downstream of the intron target site was constructed together with the intron. Upon introducing this construct into the target microorganism, an intron retrotransposition might occur in the first place, followed by homologous recombination which might result in the deletion of the target genes.

The functional annotation of operons in *Clostridium* is usually based on the comparison of *C. acetobutylicum* to *Bacillus subtilis*, whereas a significant number of predicted operons shared little homology [47]. To adequately understand the function of those unknown genes, targeted gene deletion is the first step towards identification of the function of an operon.

### Construction of CAC1493-1494 Deleted Mutant *C. acetobutylicum* DDC14

In *C. acetobutylicum*, CAC1493 and CAC1494, annotated as a Zinc finger DNA-binding domain and a hypothetical protein, are located in a two-gene operon in the chromosome. Their expression must be co-transcribed because the stop codon of CAC1493 overlaps with the start codon of CAC1494. Therefore, their biological functions are expected to be related. Zinc finger structures are found in many microorganisms and known to perform important transcriptional regulation tasks [48], [49]. We are interested in characterizing the function of CAC1493–1494, but the prerequisite is to delete this operon.

**Figure 2:**
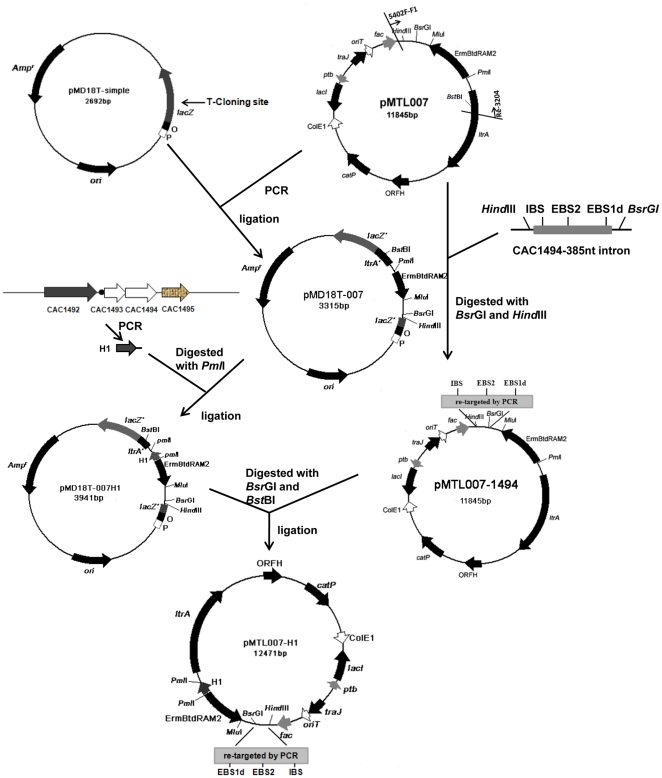
Schematic representation of the construction of pMTL007-H1 for deletion of CAC1493-1494.

**Figure 3:**
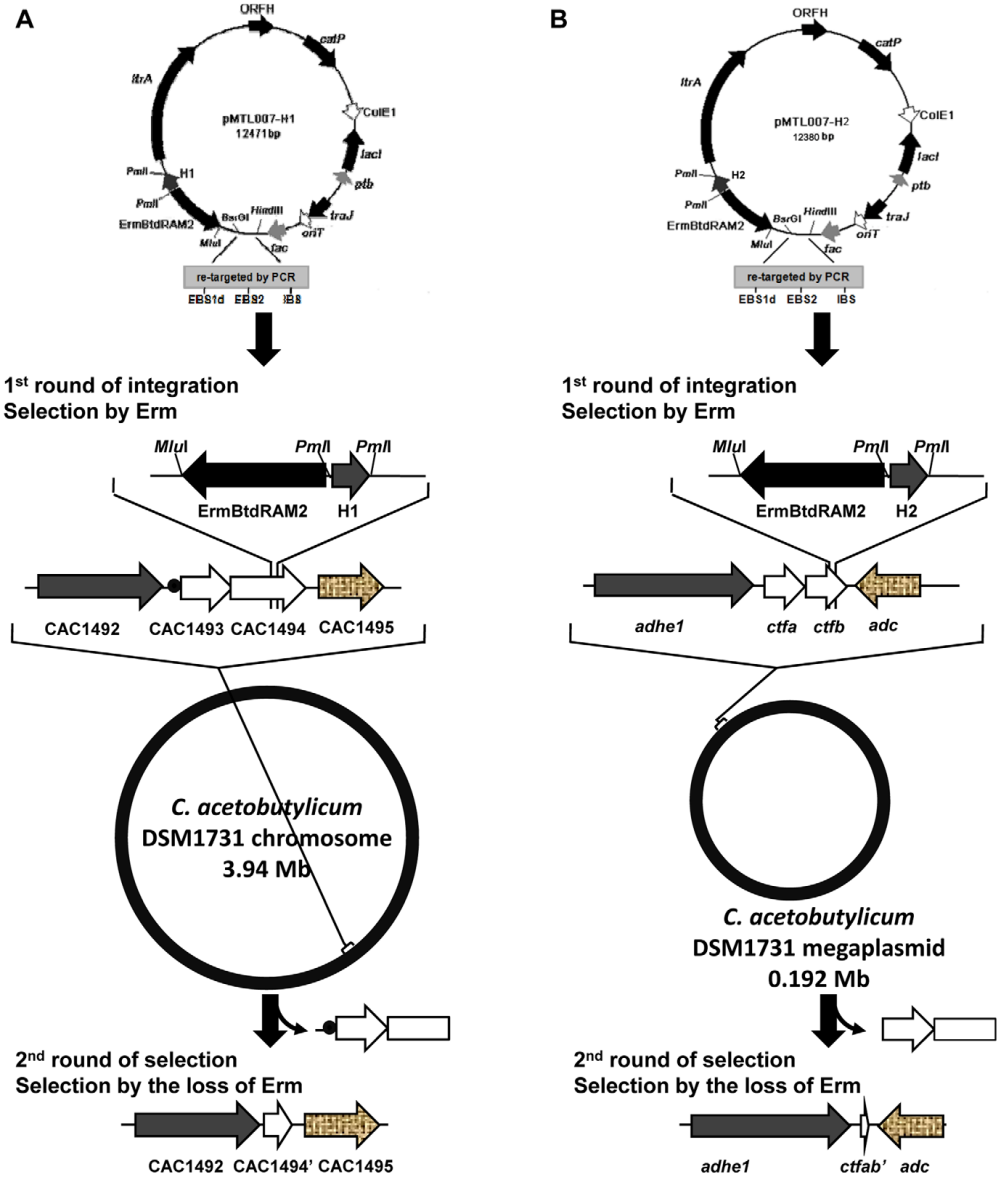
The procedure for deletion of CAC1493-1494 (A) and *ctfAB* (B) in the genome of *C. acetobutylicum*.

**Figure 4:**
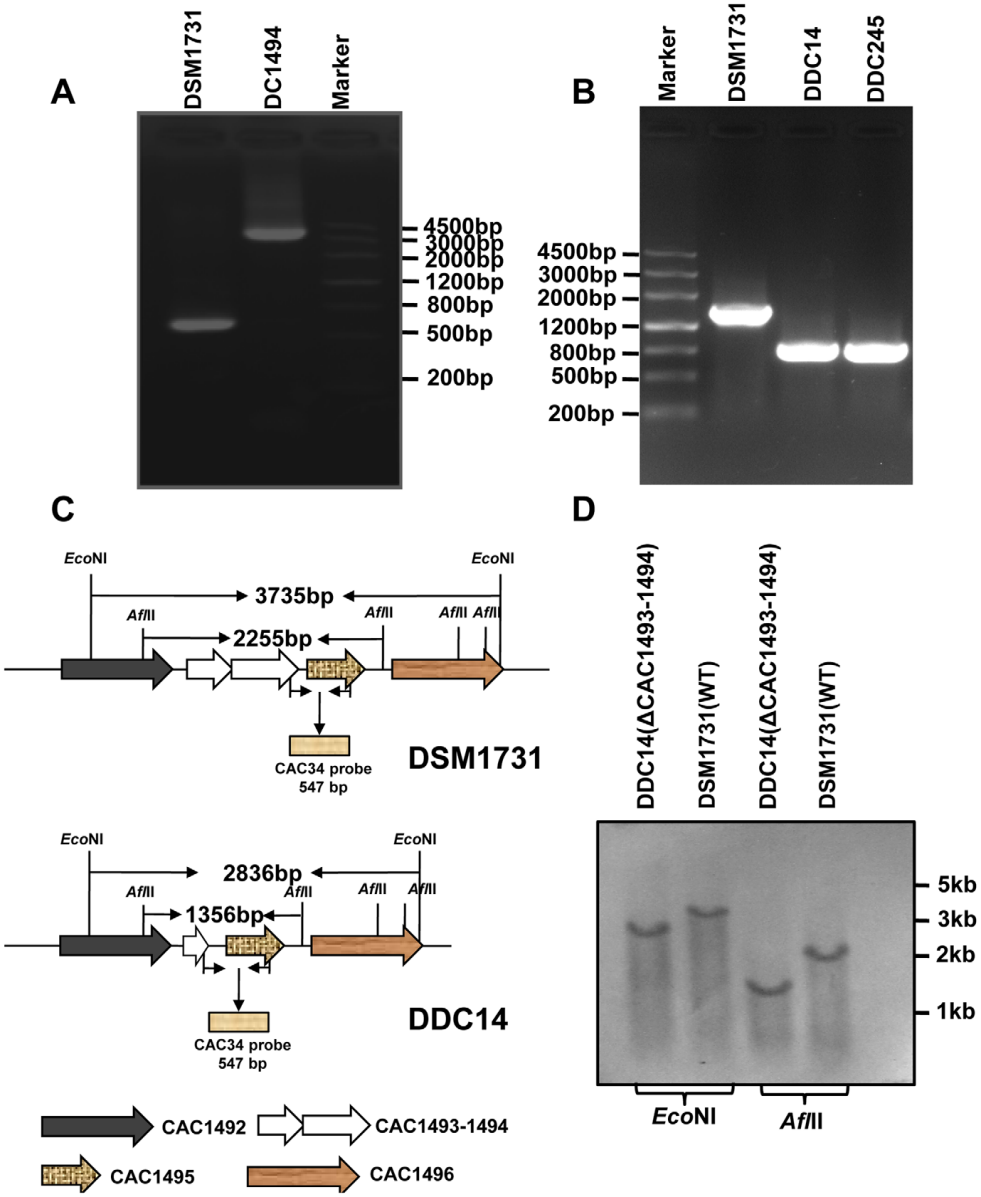
Construction of CAC1493-1494 deletion mutants. **A**. Identification of an insertion mutant by PCR using primers Cac1494B and Pex1494E flanking the target site; **B**. Identification of the deletion mutants by PCR using primers P1492-5s and Pex1494E; **C**. Schematic show of operon CAC1493-1494 and the expected deleted operon CAC1493-1494 in the chromosome; **D**. Southern blot analysis of CAC1493-1494 deletion using CAC34 probe.

To achieve an intron retrotransposition and bring a homologous allele into the genome, an intron target site in the 3′ end of CAC1494, 385/386 nt in the sense strand, was selected. Subsequently, the intron re-targeting PCR primers 1494-385/386s-IBS, 1494-385/386s-EBS1d and 1494-385/386s-EBS2 (Table 1) were designed. The DNA sequence encoding the recognition part of the intron was altered via PCR to rationally re-program intron target specificity. Finally, we modified pMTL007 by introducing the upstream fragment of operon CAC1493-1494 (626 bp, named H1) as homologous allele into the downstream region of the eythromycin resistance gene (*Erm* ^r^), the 3′-terminal sequence of intron (1907 to 2099), where multiple restriction sites including *Pml*I and *Sal*I exist (Fig. 2). Intron insertions were verified by PCR using primers Cac1494B and Pex1494E (Table 1) flanking the target site and by sequencing the PCR results. A 2.8-kb fragment containing H1, intron sequence and erythromycin resistance gene was found to be inserted into CAC1494 (Fig. 3A and Fig. 4A) with an insertion frequency of 52.9% among 51 tested clones.

**Table 1:** Bacterial strains, plasmids and primers.

Strains, plasmids or primers	Relevant characteristics	Reference or source
**Strains**		
*E. coli* Top10	*mcrA Δ*(*mrr-hsdRMS-mcrBC*) *recA1*	Invitrogen
*E. coli* JM109	*recA1 mcrB^+^ hsdR17*	Lab storage
*C. acetobutylicum* DSM1731	Contains operon CAC1493-1494, wild type	DSMZ
*C. acetobutylicum* DC1494	CAC1494::intron with H1 fragment	This study
*C. acetobutylicum* DDC14	ΔCAC1493-1494	This study
*C. acetobutylicum* DDC245	ΔCAC1493-1494	This study
*C. acetobutylicum* DCctfb3	*ctfb*::intron with H2 fragment	This study
*C. acetobutylicum* DCctfb7	*ctfB*::intron with H2 fragment	This study
*C. acetobutylicum* DDC1458	Δ*ctfAB*	This study
**Plasmids**		
pMTL007	Cm^r^, ClosTron	[24]
pMTL007-1494	Derived from pMTL007, targeting the CAC1494 in *C. acetobutylicum*	This study
pAN2	*Φ3t1*, *p15a ori*, Tet^r^, methylating DNA prior to transformation to protect it against a *C. acetobutylicum* restriction system	[24]
pMD18T-simple	Amp^r^	Takara
pMD18T-007	pMD18T-simple ligated with Ll.ltrB intron	This study
pMD18T-007H1	pMD18T-007 ligated with H1 fragment	This study
pMTL007-H1	pMTL007-1494 containing H1 fragment, CAC1493-1494 deletion vector	This study
pMTL007-ctfb	Derived from pMTL007, targeting the *ctfB* in *C. acetobutylicum*	This study
pMTL007-H2	pMTL007-ctfb containing H2 fragment, *ctfAB* deletion vector	This study
**Primer**		
5402F-F1	5′-TTAAGGAGGTGTATTTCATATGACCATGATTACG-3′	[24]
Re-3204	5′-TTCAGGTGTTATTCTTTCTGGACTTTCTCGGT-3′	This study
1494-385/386s-IBS	5′-AAAAAAGCTTATAATTATCCTTAATGGTCTATATCGTGCGCCCAGATAGGGTG-3′	This study
1494-385/386s-EBS1d	5′-CAGATTGTACAAATGTGGTGATAACAGATAAGTCTATATCATTAACTTACCTTTCTTTGT-3′	This study
1494-385/386s-EBS2	5′-TGAACGCAAGTTTCTAATTTCGGTTTCCTTTCGATAGAGGAAAGTGTCT-3′	This study
Cac1494B	5′-CGCGGATCCTTGTGTAAGCACATTTTAGG-3′	This study
Pex1494E	5′-CCGGAATTCTTATACACATATTGGCTCTC-3′	This study
P1492-5s	5′-ACGCGTCGACGCTGGTGCTTTACTTGAACT-3′	This study
Clos-5	5′-AAAACACGTGATATGGCTAAACCTCCCAAG-3′	This study
Clos-3	5′-ACGCCACGTGAAACTTGCCCTTTCCTATTC-3′	This study
Sp5	5′-AATGGTGCTGCAACAAAATATATT-3′	This study
Sp3	5′-CATCTTGATTAATAAATTCTACAT-3′	This study
CTFB572/573s-IBS	AAAAAAGCTTATAATTATCCTTATTACTTCTCACTGTGCGCCCAGATAGGGTG	This study
CTFB572/573s -EBS1d	CAGATTGTACAAATGTGGTGATAACAGATAAGTCCTCACTGATAACTTACCTTTCTTTGT	This study
CTFB572/573s -EBS2	TGAACGCAAGTTTCTAATTTCGGTTAGTAATCGATAGAGGAAAGTGTCT	This study
Dadhe-5	AAAACACGTGTCAGAACACAATATTCCTAG	This study
Dad-3	ACGCCACGTGCAATCATAATTGTCATCCCA	This study
Ct-5	AGCCAATTGGATTGTTCCTG	This study
CTFB-3	CAGCCATGGGTCTAAGTTCA	This study
Pro2-5	ACTAGATGATCAATGCACAG	This study
Pr2-3	GAGATTGTTTCTAGCTCTCA	This study

**Abbreviations**: Amp^r^, ampicillin resistance; Cm^r^, chloramphenicol resistance; Tet^r^, tetracycline resistance; *Φ3t1*, Φ3t1 methyltransferase gene of *Bacillus subtilis* phage Φ3t1. DSMZ, German Collection of Microorganisms and Cell Cultures, Braunschweig, Germany.

To delete CAC1493-1494 and the intron, secondary screening by successive transfer of the insertion mutant DC1494 into RCM medium was conducted in the absence of erythromycin. After 7 successive transfers in RCM medium (equivalent to 23 generations), two putative mutants with erythromycin sensitive phenotype were obtained from 648 colonies and designated as DDC14 and DDC245 (Fig. 3A). The genotype of the two mutants was confirmed by PCR and sequencing. Sequencing of the PCR products showed that about 0.9-kb fragment of the operon CAC1493-1494 was deleted as expected. This includes the upstream region of initiation codon (104 bp), CAC1493 and the majority part of CAC1494 (385 bp out of the total 615 bp) (Fig. 3A, 4B). Mutant DDC14 was selected for further analysis.

### Southern analysis of the deletion of CAC1493-1494

Total genomic DNA of the mutant DDC14 and the parental strain DSM1731 were isolated for Southern hybridization. DNA was digested with either *Eco*NI or *Afl*II and then probed with the labeled 547 bp CAC34 probe. Southern hybridization analysis showed that the size of the CAC34-hybridized DNA fragments of strain DSM1731 were about 0.9 kb larger than that of mutant DDC14 (Fig. 4C, 4D), suggesting that a single-copy of CAC1493-1494 exists in the genome of *C. acetobutylicum* and it had been deleted by this strategy.

### The deletion of c*tfAB* in *C. acetobutylicum* DSM 1731

**Figure 5:**
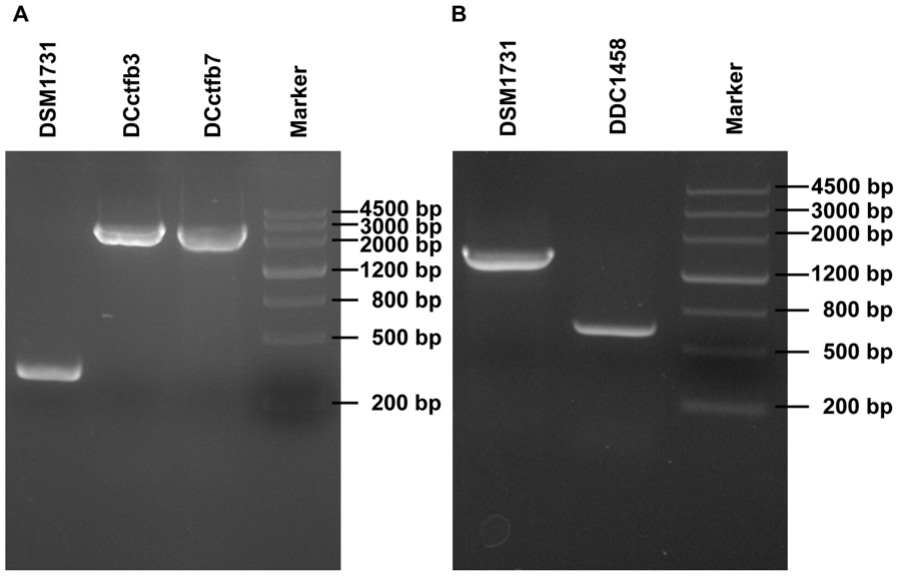
Construction of *ctfAB* deletion mutants. **A.** Identification of an insertion mutant by PCR using primers Ct-5 and CTFB-3 flanking the target site; **B**. Identification of the deletion mutant by PCR using primers Pro2-5 and Pr2-3.

To further test the applicability of this new gene deletion strategy, we selected a function known operon *ctfAB*, which is involved in acetone formation, for deletion. The reason to select this operon is because it is located in the 192-kb megaplasmid of *C. acetobutylicum*. Deletion of the operon *ctfAB* is therefore a challenge, as it is generally known that gene deletion in plasmid is more difficult. An intron target site in the 3′ end of *ctfB*, 572/573 nt in the sense strand, was selected. Subsequently, pMTL007 was modified by altering the DNA sequence encoding the recognition part of the intron and introducing a homologous allele named H2 into the downstream region of erythromycin resistance gene (Table 1). Furthermore, the intron carrying H2 was inserted into the target site, the genotype of the insertional mutants was confirmed by PCR using primers flanking the targeted site and then sequencing. A 2.5-kb fragment containing H2, intron sequence and erythromycin resistance gene was found to be inserted into *ctfB* with an insertion frequency of 82.4% among 34 tested clones (Table 1, Fig. 3B, 5A). After 10 successive transfers in RCM medium in the absence of erythromycin (equivalent to 33 generations), a *ctfAB*-deleted mutant was screened from 1998 clones and designated as DDC1458. The genotype of DDC1458 was also verified by PCR using primers Pro2-5 and Pr2-3 and sequencing (Fig. 5B). PCR sequencing results showed that a DNA fragment of 1161 bp in the operon *ctfAB* (588 bp out of the total 657 bp in *ctfA* and 573 bp out of the total 666 bp in *ctfB*) was deleted.

## Discussion

Gene deletion in microorganisms is usually conducted by homologous recombination. However, it is difficult to delete genes in *Clostridium* as the genomic integration remains a challenge [24], [36], [46]. Group II intron has been widely applied in directed insertional inactivation of a gene in many microorganisms. In *Clostridium*, after RAM was introduced into intron DIV, the intron insertion frequencies varied in the range of 1–10^−7^ integrants per cell, which is equivalent to hundreds or more Erm^R^ integrants per experiment [24]. This indicated that ClosTron had overcome the difficulties in clostridial genomic integration. Since an additional DNA fragment of 1.0 kb besides a RAM can be inserted into the DIV and the activities of RNP remain high, the DNA fragments could be those homologous to upstream or downstream region of the intron target site, which may result in gene deletion in *Clostridium* via homologous recombination [24], [30], [46], [50]. Using this approach, we successfully deleted two operons CAC1493-1494 and *ctfAB* located in the chromosome and the megaplasmid, respectively. We found that once the intron carrying the homologous fragments was inserted into the genome, it is reliable to obtain the gene deletion mutants. This suggests that the first-step integration via the retrotransposition of intron is very important to increase the chance for the following homologous recombination.

Insertional mutation in target genes by the intron has been proved highly efficient and effective [24], [30]. Jiang et al reported that successively transferring the TargeTron-inactivated *adc* mutant in antibiotic-free CGM (Clostridium growth medium) for about 100 generations, the 1.5-kb intron fragment inserted into the *adc* gene was found to be lost in about 10% of the colonies, indicating the instability of intron insertion [51]. This problem does not exist in our strategy because the inserted intron was deleted together with the majority part of the target operons via homologous recombination. Previously, the deleted genes in Clostridia, namely *spo0A* in *C. acetobutylicum*, as well as *pyrF* and *pta* in *C. thermocellum*, are all chromosomally-encoded [36], [37]. Our practice of deleting *ctfAB* provides the first example of targeted gene deletion in the megaplasmid of *C. acetobutylicum*, without affecting the megaplasmid stability, further demonstrating the effectiveness of this gene deletion strategy.

In summary, the approach described above combines the advantage of the intron retrotransposition and homologous recombination. It eliminates the selection marker during the process of negative screening, which facilitates the genetic manipulation of multiple genes and operons in both chromosome and megaplasmid. To date, group II intron was used to inactivate genes in at least ten different bacterial species [52], for most of which targeted gene deletion is impossible. Therefore, the approach developed in this study has the potential to be applied for gene deletion in those species where first-step insertion via intron retrotransposition has been established.

## Materials and Methods

### Bacterial strains, plasmids and primers

The bacterial strains, plasmids and primers used in this study are listed in Table 1.

### Growth conditions and maintenance of strains

*E. coli* strains were grown aerobically at 37°C in L broth. *C. acetobutylicum* were grown anaerobically at 37°C in reinforced clostridial medium (RCM) for routine growth and making competent cells [1], [53]. In all experiments, growth in liquid medium was monitored by measuring the absorbance at 600 nm (*A*_600_) of appropriate dilutions with a UV/Vis 2802PC spectrophotometer (Unico, New Jersey, USA). For recombinant strains, antibiotics were added to the medium at the following final concentration: 100 µg/ml for ampicillin, 30 µg/ml for chloramphenicol, 40 µg/ml for tetracycline and 25 µg/ml for erythromycin. All *C. acetobutylicum* and *E. coli* strains were stored at −80°C in RCM and L broth supplemented with 15% glycerol, respectively.

### DNA isolation and manipulation

Total genomic DNA of *C. acetobutylicum* and the plasmid DNA of *E. coli* were prepared using an E.Z.N.A Bacterial DNA Isolation Kit and E.Z.N.A Plasmid Extraction Kit (Omega Biotek Inc., Guangzhou, China). DNA restriction and cloning were performed according to standard procedure [54]. DNA sequencing were performed by Invitrogen Biotechnology Co., Ltd. Restriction enzymes, T4 DNA ligase, LongAmp *Taq* DNA polymerase, PrimerSTAR HS DNA polymerase and *Taq* DNA polymerase were purchased from New England BioLabs (Beijing, China) and TaKaRa Biotechology Co., Ltd (Dalian, China), respectively.

### Modification of pMTL007

To improve CAC1493-1494 deletion rate, ClosTron gene knockout system was employed to insert a DNA fragment as homologous allele at the target site in CAC1494 for homologous recombination. To introduce this system into CAC1494, target site for insertion was predicted and the intron re-targeting PCR primers 1494-385/386s-IBS, 1494-385/386s-EBS1d and 1494-385/386s-EBS2 were designed in line with computer algorithm available at the Sigma-Aldrich website (www.sigmaaldrich.com/TargeTron Gene Knockout) [55]. 353 bp PCR product containing the modified IBS, EBS1d and EBS2 sequences was amplified and assembled by using one-tube SOEing PCRs and then cloned into the *Hind*III and *Bsr*GI sites of pMTL007, generating pMTL007-1494 [1], [24]. Because two *Pml*I restriction sites exist in pMTL007 and H1 cannot be integrated into it directly, Ll.ltrB intron was amplified by PCR using pMTL007 as a template and 5402F-F1 and Re-3204 as primers, and then the PCR-generated fragment was TA cloned into pMD18T-simple, yielding plasmid pMD18T-007. With total genomic DNA of DSM1731 as template, primers clos-5 and clos-3 were used to amplify the homologous allele H1. Digested with *Pml*I, H1 was cloned into pMD18T-007 to generate plasmid pMD18T-007H1. Finally, a fragment containing Ll.ltrB intron and H1 was reintroduced into pMTL007-1494 through *Bsr*GI and *Bst*BI digestion, yielding plasmid pMTL007-H1 (Fig. 2).

### Electrotransformation and Screening for CAC1493-1494 deletion strains

pMTL007-H1, together with pAN2, was first transformed into *E. coli* Top10. After overnight culture, pMTL007-H1 was isolated and then electroporated into *C. acetobutylicum* according to the protocol developed by Mermelstein[56]. The insertion mutants were screened based on the protocol reported by Heap [24], Intron insertions were verified by PCR using primers Cac1494B and Pex1494E flanking the target site and then PCR products were purified and sequenced, the sequence has been deposited in (Genbank number: HQ257448).

The insertion mutant DC1494 was inoculated into RCM without erythromycin resistance for successive transfer culture. For successive transfer, one milliliter fully grown culture was inoculated into 10 ml fresh RCM medium, grown anaerobically at 37°C for 12 h until full growth achieved. This transfer process was repeated for at least 7 times, each transfer is equivalent to three generations. The resulted culture was plated onto RCM plates containing 1.5% (w/v) agar. Isolated colonies were tested for the ability to resist erythromycin by streaking them onto solid RCM supplemented with 25 µg/ml erythromycin. The colonies without erythromycin resistance will be verified by Colony PCR using primers P1492-5s and Pex1494E (Fig. 3A). The PCR products were sequenced and the sequence has been deposited in (Genbank number: HQ257447).

### Southern blot analysis of CAC1493-1494 deletion

For Southern blot analysis of CAC1493-1494 deletion, the genomic DNA from the wild-type strain DSM1731 and mutant DDC14 was extracted, digested with *Eco*NI and *Afl*II, separated by electrophoresis overnight on a 0.8% agarose gel, and then transferred onto a hybond-N membrane in 0.4N NaOH. Hybridization was performed with digoxigenin-labeled DNA probe using DIG High Prime DNA Labelling and Detection Starter Kit I (Roche Diagnosis GmbH, Roche Applied Science, 68298 Mannhein Germany). DNA probe CAC34, amplified from the reserved region of CAC1494 and its downstream fragment with primers sp5 and sp3, were generated by random primed labeling technique according to the manual of the manufacturer.

### The deletion of c*tfAB* in *C. acetobutylicum* DSM 1731

The deletion of *ctfAB* was based on the above mentioned strategy (Fig. 1). The DNA sequence encoding the recognition part of the intron in pMTL007 was altered by PCR using primers CTFB572/573s-IBS, CTFB572/573s -EBS1d, CTFB572/573s -EBS2, yielding plasmid pMTL007-ctfb. The homologous allele H2 of 535 bp was amplified by PCR using primers Dadhe-5 and Dad-3, cloned into pMD18T-007, and then ligated into vector pMTL007-ctfb after *Pml*I digestion, generating plasmid pMTL007-H2. The electroporation of pMTL007-H2, the retrotransposition of intron carrying the homologous allele at the site of 572/573 nt in *ctfB* and the deletion of *ctfAB* followed the above mentioned methods (Fig. 2 and Fig. 3B). The genotype of the insertion or deletion mutants was confirmed by PCR using primers flanking the targeted site or region and then by sequencing (Genbank number: HQ683763, 683762) (Fig. 5).
